# Prevalence and predictors of help-seeking for women exposed to spousal violence in India – a cross-sectional study

**DOI:** 10.1186/s12905-017-0453-4

**Published:** 2017-11-03

**Authors:** Malin Leonardsson, Miguel San Sebastian

**Affiliations:** 1 Umeå International School of Public Health, Umeå University, Umeå, Sweden; 2 Department of Public Health and Clinical Medicine, Epidemiology and Global Health, Umeå University, Umeå, Sweden; 3 The Swedish Board of Student Finance, Sundsvall, Sweden

**Keywords:** Spousal violence, Help-seeking, Prevalence, Predictors, India, Ecological model

## Abstract

**Background:**

Spousal violence against women is prevalent in India (29%). Studies from various countries have shown that few women exposed to intimate partner violence or spousal violence seek help, especially in low-income countries. The objective of this study was to estimate the prevalence and predictors of help-seeking among women in India who have experienced various types of spousal violence.

**Methods:**

Cross-sectional data on 19,125 married, separated, divorced or widowed women in India who had experienced physical or sexual violence at the hands of their husbands were obtained from the India National Family Health Survey III 2005–2006. Bivariate and multivariate logistic regression analyses were carried out.

**Results:**

Less than one fourth (23.7%) of married, separated, divorced or widowed women in India who had experienced some form of physical or sexual spousal violence had sought help, but only 1% had sought help from formal institutions. Help-seeking was most prevalent in women who had been exposed to a combination of physical, sexual and emotional abuse (48.8%) and the least prevalent in women who had experienced sexual violence only (1.5%). Experience of severe violence and violence resulting in injury were the strongest predictors of help-seeking. Having education, being Christian or an acknowledged adherent of another minority religion - mainly Buddhism and Sikhism (Islam not included), getting married after the age of 21 and living in the South region were also associated with seeking help. Women in the North and Northeast regions were less likely to seek help, as were women with children and women who thought that a husband could be justified in hitting his wife.

**Conclusions:**

Very few Indian women who experience spousal violence seek help. The characteristics of the violence are the strongest predictors of help-seeking, but sociodemographic factors are also influential. We recommend efforts to ensure educational attainment for girls, prevention of child marriages, and that police officers and health care staff should be educated about intimate partner violence and in how to respond to women who seek help. It is important to tackle norms and attitudes surrounding violence against women, as well as attitudes to women who disclose violence.

**Electronic supplementary material:**

The online version of this article (10.1186/s12905-017-0453-4) contains supplementary material, which is available to authorized users.

## Background

Intimate partner violence (IPV) is a worldwide public health problem. It is estimated that 30% of all ever-partnered women have been exposed to physical or sexual IPV at least once in their life [1] and the vast majority of the women who are exposed to physical violence experience repeated violence [2]. Health consequences of IPV include self-reported poor health, pain, difficulties with walking, problems in performing daily activities, dizziness [3] and reproductive health problems like miscarriages and induced abortions [2]. Being exposed to violence from a partner is also associated with mental health problems such as memory loss, concentration problems, feelings of worthlessness, suicidal thoughts [4] and an overall lower quality of life [5]. IPV is prevalent in all societies and in all socio-economic groups, but the highest prevalence is found in the South-East Asia region [1]. This study focuses on women in India who have been exposed to violence by their husband.

Since the 1970s there has been growth in research on IPV and spousal violence [6]; recently there has been increasing interest in women’s help-seeking behaviour. Research suggests that social support is associated with a lower risk of further violence over a one-year period [7], lower incidence of depression and higher self-esteem [8]. Also, a recent study from Bangladesh showed that women who sought help from legal institutions experienced less violence from their husband [9]. Yet many women exposed to IPV do not seek help. The World Health Organization (WHO)‘s multi-country study of IPV reported that 55–95% of women who had experienced physical or sexual IPV have never sought help from formal institutions [2]; however the prevalence of help-seeking and disclosure varies widely between countries. In New Zealand 77% of female victims of physical IPV and 61% of victims of sexual IPV have told someone about the violence [10] but in many other countries the majority of women who are exposed to IPV or spousal violence remain silent about it. In Pakistan and Bangladesh only 35% and 33% respectively have disclosed their experience of violence [11, 12]. In Tanzania and Jordan help-seeking rates for IPV were around 40% [2, 13] while 24–26% of women in India who have been exposed violence from their husband seek help from someone [14–16]. The proportion of women who seek help from formal institutions is usually lower, 22% in Serbia [17], less than 6% in Jordan [13] and only 2% and 1% respectively in Bangladesh [12] and India [14, 15]. Studies show a clear pattern; women in low income countries and in countries with large gender inequalities and rigid gender roles seek less help than women in countries with higher levels of gender equality where gender roles are less strict.

### IPV in India

Fundamental to understanding men’s violence against women, and women’s help-seeking behaviour, is gender power relations. In South Asia gender roles are rigid and there are widespread, deep-rooted patriarchal values that emphasise male authority in several aspects of everyday life. Patriarchal culture enforces male dominance and entitlement to control [18] and violence is used to control female obedience [19] and to disciplining women [20]. In many parts of South Asia the police force, legal departments and health sectors are permeated with patriarchal norms and values, which make help-seeking difficult for women [18].

There is a widespread acceptance of violence against wives in India, which is demonstrated by the results of a nationally representative survey conducted in 2005–2006. It showed that 54% of women in India thought that a husband is justified in hitting or beating his wife in at least one of these situations: a) the wife goes out without telling her husband; b) the wife neglects the house or the children; c) the wife argues with her husband; d) the wife refuses to have sex with her husband; e) the wife does not cook properly; f) the husband suspects his wife of being unfaithful or d) the wife is disrespectful to her in-laws). Half of Indian men (51%) also endorsed wife-beating on these terms [21]. Based on data from the National Family Health Survey III 2005–2006 (NFHS-3), which included nearly 67,000 married, separated or divorced women, Kavitha (2012) concluded that 35% of married, separated, divorced or widowed women in India had experienced physical violence from their husband during their marriage; 16% had experienced emotional abuse and 10% had experienced sexual violence [16].The most recent NFHS from India (NFHS-4 2015–2016) shows that prevalence of spousal violence in India has declined to 29% [22].

Prior to 1983, India did not have any legal regulations on violence within marriage [19]. The Protection of Women from Domestic Violence Act (PWDVA) was passed in 2006. The Act was intended to provide more effective protection for women who are victims of any kind of violence within the family [23], but according to Ghosh and Choudhuri (2011) it has failed to address domestic violence and there is large regional variation in implementation, in for example how the police record crimes. Also, awareness of the PWDVA is lower among rural populations than among urban ones. Women sometimes face lengthy and costly legal processes, which can be discouraging to report violence. There are also cases where delays in implementing Protection Orders have contributed to exposing the victims to more violence. Factors that have contributed to the failure of the Act are, according to Ghosh and Chouduri, lack of guidelines to judges and an apathetic and negative role of the (sometimes corrupt) police. A third contributor to the failure is the apathy of the society in general. The Indian society tends to view violence within the household as a private affair [24]. To this date marital rape is not a crime in Indian legislation.

### Barriers to help-seeking

The discourse on IPV in South Asia is victim-blaming and focuses on what abused women have done to cause her husband to be violent and why she does not tolerate violence within marriage. Women are socialized into feeling responsible for family integrity and for solving relationship problems. This promote self-blame and make women feel responsible for the violence, which in turn affect their self-esteem and help-seeking behaviours. There is also stigma attached to seeking help for spousal violence and women are socialized into feeling shame and guilt if disclosing abuse [18].

Evidence from interviews with perinatal women in Mumbai who had recently experienced spousal violence revealed that fear of social repercussions, fear of escalated violence and fear that the husband would demand divorce were reasons for not disclosing violence. Some expressed the view that there were no real options, and the dilemma of being economically dependent on their husband for a living. Others described abuse as a normal part of marriage for women. Some women had negative experiences from seeking help from formal services, for example women been told by the police that the violence she was exposed to was a private issue between the husband and wife [25]. A study from Northern India on community member’s perceptions of options for women exposed to spousal violence found that seeking help from formal institutions was seen as both infeasible and inappropriate. Participants perceived that an abused woman who seeks help from the police will be told to modify her behaviour, something that some participants themselves agreed with. Others were scared of the police because they did not know much about them [26].

In Bangladesh some women do not disclose violence because they think that the husband has the right to use violence against his wife, while other reasons include fear of jeopardizing the family’s honour, stigma and fear of threats of murder [12]. Thinking that the violence is not so serious or even normal, self-blame, fear of being blamed and hoping that their partner will change are some of the reasons why Serbian women do not seek help [17]. In addition, evidence from the United States show that lack of knowledge about support services, the perception that seeking help from formal institutions will not be useful, fear of losing housing and lack of money can be obstacles for seeking help from formal institutions [27]. The latter shows that women of low socioeconomic status and women who are economically dependent on their husbands also face challenges related to financial aspects. This obstacle is likely to be more evident in societies with poor social safety nets. For example, participants in a study from Kenya talked about economic dependence on their husband as a major obstacle to seeking help for spousal violence [28].

Women in India who seek help for spousal violence mainly turn to their own family for support, while their husband’s family and neighbours are the second and third most commonly approached sources for help [16]. A survey of 1038 women in clinics in the slums of Mumbai indicated that 67% would be willing to disclose violence by their husband if they were asked about it in a health care setting. Less than 5% had actually been asked such questions [25].

### Factors associated with help-seeking

Research from different contexts have found that factors that are strongly associated with a higher probability of seeking help for physical IPV include experience of repeated violence [29, 30] or severe violence [12, 17, 29, 30]. Suffering injury as a consequence of violence is associated with seeking help from the police or medical services [31]. A number of socio-demographic factors have also been associated with seeking help. In Mexico women of very low socioeconomic status has been reported to be less likely to seek help than women of low socioeconomic status [32], while education and living in urban areas has been positively associated with help-seeking from formal institutions [29, 32]. In Pakistan having at least some formal education, having an independent income and living in a ‘non-overcrowded’ household increased the probability of disclosing physical spousal violence [11]. Other factors that have been associated with disclosure or seeking help for IPV include youth, thinking that a man hitting a woman can be justified [11], presence of children [30] and religious affiliation [33]. However, some studies have found that for example age, religion [12], education and socio-economic status [13] are not significant predictors of help-seeking or disclosure of IPV.

A study from Nigeria examined individual as well as contextual factors associated with seeking help for sexual and physical violence against women. The data suggested that few individual-level factors were related to seeking help although a number of contextual factors, such as living in Nigerian states with a lower Human Development Index or higher incidence of violence, were negatively associated with seeking help. Women who had witnessed their father beat their mother had higher odds of seeking help for physical and sexual violence [34].

A study on women’s help-seeking for gender-based physical and sexual violence in 24 low and middle income countries, indicated that India had a very low prevalence of help-seeking from formal institutions for such violence compared to many other countries in the study. Only 1% sought help from formal institutions when exposed to gender-based violence. Around 32% sought help from someone. The likelihood of seeking help from formal institutions in India increased with age, while having no education and living far from a health care facility was associated with less help-seeking [35].

Many studies of IPV in India have assessed the prevalence and risk factors for IPV [19, 36–40] but there have been few studies addressing the predictors of help-seeking for IPV in Indian women. Knowledge of the factors which are associated with seeking help for IPV in India is necessary to address the problem and plan interventions. Till recently no comprehensive study in India regarding factors determining whether an individual seeks help, whether formal or informal, for IPV or spousal violence existed. However, while the current study was under review, three studies with a similar approach and using the same database were published. Paul (2016) examined the extent to which sociodemographic differences among women impacted their participation in both informal and formal help-seeking behaviours using a different conceptual framework to ours [15]. The findings indicated that education and employment were the two most important sociodemographic determinants for seeking help from both formal and informal sources. Rowan et al. (2015) focused on the role of female empowerment for formal and informal help-seeking after spousal violence using individual-, relationship- and state-level measures of empowerment. They found that severe violence and injury from violence were the strongest correlates of seeking help and that overall, individual-level factors and measures of empowerment were not related with help-seeking. However, living in states with higher scores in gender empowerment measure and having a husband who exerted a greater number of controlling behaviours were factors that increased the odds of seeking help [14]. The latter finding is supported by Hayes and Franklin’s study (2016) using the same database. Apart from individual- and relationship level factors, such as employment, increased decision-making and severe violence, Hayes and Franklin also found significant predictors for help-seeking at an aggregated level. As the age of marriage for women in a community increased, the odds of help seeking also increased. In addition, a high proportion of women who experience severe and/or sexual violence in a community was negatively associated with help-seeking [41].

Our study had two related objectives: i) to estimate the prevalence of help-seeking in women in India who experience different types of spousal violence and ii) to identify the demographic, socioeconomic, social and psychological factors which predict whether women who experience various types of spousal violence will seek help.

## Methods

### Database

This study was based on the Indian NFHS-3. This was a survey of a nationally representative sample, conducted between December 2005 and August 2006 through face-to-face interviews based on questionnaires. The women’s questionnaire was administered to 131,596 women aged 15–49 years; 124,385 women completed the interviews. The section on domestic violence was administered to 69,484 of the 93,724 women who reported that they were married, separated, divorced or widowed. Only one woman in each household was interviewed about domestic violence in order to allow the respondent to keep the information confidential. If there were more than one eligible woman in a household, one was randomly selected for the questions on domestic violence. Because of the sensitive nature of the questions the respondents were interviewed by trained female field workers. Interviews took place in the women’s homes but the questions on domestic violence were only asked if privacy could be obtained. Less than 1 % of the women administered to the domestic violence section could not be interviewed because privacy could not be ensured. For further details about the data collection see International Institute for Population Sciences and Macro International’s publications on the NFHS-3 [42, 43]. After receiving permission from The Demographic and Health Surveys (DHS) Program the NFHS-3 data were downloaded from their website (http://www.dhsprogram.com) in STATA format.

### Definition and measurement of IPV and spousal violence

There is no consensus definition of IPV but it is often referred to as behaviour by an intimate partner that causes physical, sexual or psychological harm to the other partner [1]. The definition of ‘intimate partner’ often includes partners in any romantic relationship or partners living together, but some researchers have limited their work to consideration of violence by spouses or former spouses [44]. Since only women who were or had been married were asked detailed questions about physical, sexual and emotional violence in NFHS-3 we have used the term ‘spousal violence’ in this study.

NFHS-3 data on physical, sexual and emotional abuse were based on the responses to the following questions:

Physical spousal violence: *(Does/did) your (last) husband ever do any of the following things to you:*

- *Slap you*
- *Twist your arm or pull your hair*
- *Push you, shake you, or throw something at you*
- *Punch you with his fist or with something that could hurt you*
- *Kick you, drag you or beat you up*
- *Try to choke you or burn you on purpose*
- *Threaten or attack you with a knife, gun, or any other weapon*
- Sexual spousal violence: *(Does/did) your (last) husband ever do any of the following things to you?*
- *Physically force you to have sexual intercourse with him even when you did not want to*
- *Force you to perform any sexual acts you did not want to do*

Emotional spousal abuse: *(Does/did) your (last) husband ever:*

- *Say or do something to humiliate you in front of others?*
- *Threaten to hurt or harm you or someone close to you?*
- *Insult you or make you feel bad about yourself?*

Only married, separated, divorced or widowed women who indicated in responses to the domestic violence section of the questionnaire that they had only experienced violence from their husband were included in this study. The question about seeking help related to all the respondent’s experiences of physical and sexual violence, so to be certain that responses related to spousal violence we excluded data from women who had experienced physical or sexual violence from someone other than their husband (5424 observations). Women who had not answered all the questions relating to whether they had experienced physical or sexual violence at the hands of someone other than their husband were also excluded (11 observations). Data from women who had not answered the questions about help-seeking (341 observations) and women who had not answered the questions about physical and sexual spousal violence (23 observations) were excluded. The final sample in this study consisted of 19,125 women who had all been exposed to physical and/or sexual violence by their current or former husband. 6742 of these women had also experienced emotional abuse.

We categorized women in terms of their experience of physical and sexual spousal violence and spousal emotional abuse, on the basis of their responses to the relevant questions, before estimating the prevalence of help-seeking in each group. The groups were as follows i) women who had experienced some form of spousal violence (all 19,125 women in this study); ii) women who had experienced physical violence only; iii) women who had experienced sexual violence only; iv) women who had experienced both physical and sexual violence; v) women who had experienced physical violence and emotional abuse; vi) women who had experienced sexual violence and emotional abuse and vii) women who had experienced all three types of violence or abuse. Only women who had experienced physical or sexual violence were asked the questions about help-seeking, so none of the women in the sample had experienced emotional abuse only.

### Dependent variable

The dependent variable was binary: individuals were classified as having sought help from someone or not having sought help. The survey question used to derive data on help-seeking was: *Thinking about what you yourself have experienced among the different things we have been talking about, have you ever tried to seek help to stop the person(s) from doing this to you again?* (yes/no).

We derived the data on the source women turned to for help from the question *From whom have you sought help to stop this?* Potential sources of help included both informal and formal sources. Informal sources included the woman’s own family, her partner’s or husband’s family, friends, neighbours and current or former boyfriends. Formal institutions included the police, criminal justice system, health care staff, social services, religious leaders and other persons. Because the number of women who had sought help from formal institutions was very small (see Table 1) no distinction was made between formal and informal sources of help in the logistic regression analyses.

### Independent variables

The choice of independent variables was based on Heise’s (1998) ecological model of violence against women. The ecological model recognises four levels of analysis: personal history, the micro-system, the exo-system and the macro-system [45]. The first level, personal history, encompasses personal characteristics and life history [46]. Witnessing violence between one’s parents in childhood [45], mental health [46] and education [18] are dealt with at this level. The second level, the micro-system, includes family-level factors and the woman’s relationships with family and friends. Factors such as the presence of children, family norms [46], the extent to which the husband controls the family’s wealth, verbal conflicts in the household and the structure of the traditional family are dealt with at this level [45].

The third level, the exo-system, encompasses environmental factors and social structures, such as the neighbourhood and community in which the family lives, its socioeconomic status and whether the woman is employed or not [45]. Lack of access to resources and support systems can be related to the exo-system [18]. Finally, the fourth level, the macro-system, represents the broader cultural context in which the woman and her family and community live. Cultural beliefs and values that pervade the other three levels, such as rigid gender roles [45] and societal acceptance of IPV [18], are central factors.

The personal history variables included in this study were age (15–19; 20–24; 25–29; 30+) [12], age at first marriage (<18; 18–20; 21+) [19], education level (not completed primary education; completed primary education; completed secondary education or higher) and whether the woman’s father had ever beaten her mother (yes/no). Most variables in the present study were categorized based on categorizations in other studies using the same population data or equivalent population data from neighbouring countries. Secondary education and higher education were merged into one category because of a small number of women who had completed a higher education level than secondary school in the analyses of predictors of help-seeking for specific types of violence. Two variables related to violence were generated and classified as personal-level factors: severity of violence and violence resulting in injury. The severity of the physical violence to which the respondent had been subjected by her husband was categorized as ‘moderate’ or ‘severe’ using the WHO criteria [1]. Moderate violence includes being slapped, having one’s arm twisted or hair pulled, being pushed or shaken or having something thrown at one. Severe violence includes being punched with a fist or other object, being kicked, dragged, beaten up or choked, being burnt on purpose or being threatened with a weapon of any kind. The injury variable was a binary variable capturing whether or not the respondent had ever had any physical injuries as a consequence of her husband’s violence. When possible, we allocated the variables to different system-levels of the ecological model based on other published literature [18, 45, 46]. Severity of violence and Injury, two variables not found in the literature on the ecological model, were classified as personal history factors because we considered them to be part of what a victim of spousal violence has experienced (or not experienced).

Duration of marriage (0–4; 5–9; 10–19; 20+) and number of living children (0; 1–2; 3–4; 5+) [19] were the two micro-system variables included in this study. They were classified as micro-system because they were directly related to the family. The exo-system variables were place of residence (dichotomous: urban; rural), wealth index (poorest; poorer; middle; richer; richest), woman’s occupation (no paid occupation; agricultural sector; other occupation - mainly skilled and unskilled manual labour) and caste or tribal identity. Caste or tribal identity was categorized as Scheduled caste (SC), Scheduled tribe (ST), Other backward class (OBC) and Others (including other caste groups, women who did not belong to any caste and women who did not know to which caste they belonged) [47]. SC and ST are considered the lowest castes in India and are the most socially disadvantaged; OBC is regarded as an intermediate caste [48, 49]. Socioeconomic status and whether the woman is employed or not are exo-system variable in Heise’s study [45] and was therefore, together with caste, also allocated to the exo-system level in our ecological model. The macro-system variables, that represent the broader cultural context in this study were region (North; East; Northeast; West; Central; South [16, 19]) and religion (Hindu; Muslim; Christian; Other - mainly Buddhist or Sikh [19]).

Finally, a variable for attitudes to violence was included. Respondents were asked if they thought that a husband is justified in hitting or beating his wife in seven different situations (the wife goes out without telling her husband; the wife neglects the house or the children; the wife argues with her husband; the wife refuses to have sex with her husband; the wife does not cook properly; the husband suspects his wife of being unfaithful or the wife is disrespectful to her in-laws). There were two response categories, No (wife-beating is never justified in any of these situations) and Yes (wife-beating is justified in at least one of these situations) [13]. Although a woman’s attitude to wife-beating can be regarded as a personal characteristic, attitudes towards violence against women can shaped by factors at all levels of the social order [50]). Witnessing one’s father use violence against one’s mother is an example of personal history that can influence what attitude a woman has towards IPV. Exo-system variables that can influence attitudes are labour market participation and socioeconomic status, while attitudes are also constructed by larger cultural contexts and factors such as mass media and laws [50]. The variable Thinks that violence can be justified was therefore included in the analysis as a fifth, ‘trans-system’ factor in our ecological model.

### Statistical analysis

The data were analysed using STATA version 13 statistical software. Since the objective was to achieve representativeness at the national level, the NFHS-3 data were weighted using the national domestic violence weight variable (D005S) according to standard procedure [43]. The ‘svy:’ command was used when generating descriptive statistics and conducting logistic regression analyses to take account of survey weighting.

Frequency tables for the distribution of respondents into non-help-seeking and help-seeking categories in terms of the independent variables were generated. The overall prevalence of help-seeking and prevalence with which help was sought from formal institutions were calculated as a function of type of violence or abuse.

Factors associated with seeking help for spousal violence were analysed by running bivariate logistic regression including one independent variable at a time to obtain the crude odds ratios for each variable. Next multivariate logistic regression adjusting for all other variables was run. Separate series of bivariate and multivariate logistic regressions were also run for the various exposure groups. Two exposure groups were small samples (sexual violence only: 811; sexual violence combined with emotional abuse: 155) and only small subsets of these women had sought help (13 and 8 respectively; see Table 1). Therefor no logistic regression analyses were run for these two groups. We mainly focused on the predictors of help-seeking for women who had experienced any form spousal violence but the results from the logistic regressions for the various exposure groups are presented in Additional file 1: Table S1.

Missing data and ‘don’t know’ responses for independent variables were recoded as missing values (2815 observations) resulting in 14.7% missing values in the logistic regressions.

The significance level used in all tests was α = 0.05. All variables that resulted in at least one significant crude odds ratio (*p* < 0.05) in the bivariate logistic regression were included in all multivariate logistic regressions. Factors which were not significant in a bivariate model but have been associated with help-seeking behaviour in previous research (religion; attitude to wife-beating) were also included in the multivariate logistic regressions.

## Results

### Prevalence of help-seeking

Almost a quarter (23.7%) of the 19,125 women who had experienced some kind of violence at the hands of their husband had sought some kind of help and 1.0% had sought help from formal institutions (Table 1). 17.0% of the women who had experienced physical violence only (*n* = 10,080) had sought help and 0.4% had sought help from formal institutions. Figures were similar for women who had experienced both physical and sexual violence (*n* = 1762); 22.2% and 0.4%. About a third (33.5%) of women who had experienced both physical violence and emotional abuse (*n* = 4429) had sought some kind of help; 1.4% had sought help from formal institutions.

As Table 1 indicates, women who had experienced sexual violence only were the group least likely to have sought help. Only 13 out of 811 (1.5%) had ever sought help to address the violence and none had turned to a formal institution. 8 out of 155 women who experienced both sexual violence and emotional abuse had sought help (6.2%), including one who had sought help from formal institutions (0.3%). Women who had experienced all three types of violence or abuse were most likely to have sought help, with 48.8% having done so, including 4.9% who had sought help from formal institutions.

**Table 1:** Frequencies and percent of women in India who seek help and help from formal institutions for different types of violence

Type of violence	Total (N)	Any Help			Formal help		
		*N*	%	95% CI	*N*	%	95% CI
Any violence	19 125	4 508	23.7	22.8–24.7	211	1.0	0.9–1.2
Physical	10 080	1 674	17.0	16.0–18.2	36	0.4	0.2–0.5
Physical + sexual	1 762	397	22.2	19.9–24.7	12	0.4	0.2–0.8
Physical + emotional	4 429	1 487	33.5	31.5–35.5	62	1.4	1.0–1.9
Sexual	811	13	1.5	0.8–2.8	0	0	–
Sexual + emotional	155	8	6.2	1.8–18.9	1	0.3	0.0–1.9
Physical + sexual + emotional	1 888	929	48.8	46.0–51.6	100	4.9	3.8–6.1

Table 2 compares the characteristics of women who had sought help for spousal violence with the characteristics of women who had not sought help. Women who had experienced severe violence were more likely to have sought help than those who experienced moderate violence only (39.7% and 13.4% respectively). Women who had had a physical injury as a consequence of violence were more likely to have sought than those who had not had physical injuries (41.9% and 14.1% respectively). There were also large regional differences in the prevalence of help-seeking; in the South region 33.4% of the women who experienced spousal violence had sought help whereas in the Northeast region only 17.3% had sought help.

**Table 2:** Distribution of respondents according to no help and help-seeking behaviour by factors at different levels

		No help		Help	
		*N*	%	*N*	%
Personal history					
Age	15 to 19	569	79.2	149	20.8
	20 to 24	2 184	77.3	621	22.7
	25 to 29	3 169	74.8	1 020	25.2
	30 to 49	8 695	76.2	2 718	23.8
Age at first marriage	<18	9 162	76.8	2 784	23.2
	18 to 20	3 605	76.5	1 100	23.5
	21+	1 850	72.8	624	27.2
Education	<Primary	7 924	77.1	2 363	23.0
	Primary	2 548	74.9	838	25.1
	≥Secondary	4 145	75.5	1 307	24.6
Violent father	No	9 228	76.6	2 777	23.4
	Yes	3 788	73.5	1 375	26.5
Severity of violence	Moderate	10 239	86.6	1 544	13.4
	Severe	4 378	60.3	2 964	39.7
Injuries	No	10 807	85.9	1 783	14.1
	Yes	3 773	58.1	2 718	41.9
Micro-system					
Duration of marriage	0–4	1 510	79.0	407	21.0
	5 to 9	2 965	75.6	887	24.5
	10 to 19	6 014	75.0	1 957	25.0
	20+	4 128	77.2	1 257	22.8
No. of living children	0	951	72.2	345	27.8
	1 to 2	6 049	75.6	1 936	24.4
	3 to 4	5 627	76.5	1 702	23.6
	5+	1 990	79.8	525	20.3
Exo-system					
Place of residence	Urban	5 585	75.1	1 732	24.9
	Rural	9 032	76.7	2 776	23.3
Caste or tribe	SC	3 172	73.8	1 116	26.2
	ST	1 903	77.4	535	22.6
	OBC	4 867	74.1	1 670	25.9
	Other	4 597	80.0	1 172	20.1
Wealth Index	Poorest	3 078	77.7	895	22.4
	Poorer	3 128	75.7	993	24.3
	Middle	3 209	75.8	1 049	24.2
	Richer	3 186	75.2	1 013	24.9
	Richest	2 016	77.7	558	22.4
Occupation	No occupation	7 429	78.4	1 986	21.6
	Agricultural	4 004	75.0	1 333	25.0
	Other	3 176	73.3	1 185	26.7
Macro-system					
Region	North	2 006	77.4	578	22.6
	East	2 977	80.0	727	20.0
	Northeast	2 420	82.7	488	17.3
	West	1 516	79.4	433	20.6
	Central	3 289	74.7	1 126	25.3
	South	2 409	66.6	1 156	33.4
Religion	Hindu	11 068	76.2	3 451	23.8
	Muslim	2 150	78.6	580	21.5
	Christian	787	74.3	247	25.7
	Other	593	72.6	224	27.4
Trans-system					
Thinks that violence can be justified	No	5 214	76.2	1 640	23.9
	Yes	8 747	76.3	2 672	23.7
	Total	14 617	76.3	4 508	23.7

### Predictors of help-seeking: Bivariate model

The bivariate regression (Table 3) showed that many factors were associated with seeking help for spousal violence in India. Women aged 25–29 years, women who were at least 21 years old when they married, women who had completed not higher than primary education and women whose father had beaten their mother were more likely to have sought help compared to the reference groups. The two most important bivariate relationships were the association between severe violence and seeking help (OR: 4.26; 95% CI 3.86–4.70) and between having had an injury and seeking help (OR: 4.38; 95% CI 3.97–4.83).

**Table 3:** Bivariate and multivariable logistic regression analyses for predictors of seeking help for spousal violence

		Unadjusted model		Adjusted model^a^	
Personal history		OR	95% CI	OR	95% CI
Age	15 to 19	1		1	
	20 to 24	1.12	0.88–1.42	1.08	0.81–1.44
	25 to 29	**1.28**	1.01–1.62	1.09	0.77–1.53
	30 to 49	1.19	0.95–1.48	0.96	0.67–1.40
Age at first marriage	<18	1		1	
	18 to 20	1.01	0.92–1.12	1.04	0.92–1.18
	21+	**1.23**	1.08–1.41	**1.29**	1.07–1.55
Education	<Primary	1		1	
	Primary	**1.12**	1.00–1.26	**1.17**	1.02–1.35
	≥Secondary	1.09	0.98–1.21	**1.28**	1.10–1.48
Violent father	No	1			
	Yes	**1.18**	1.07–1.31	0.99	0.88–1.10
Severity of violence	Moderate	1		1	
	Severe	**4.26**	3.86–4.70	**2.80**	2.50–3.15
Injuries	No	1		1	
	Yes	**4.38**	3.97–4.83	**2.79**	2.49–3.14
Micro-system					
Duration of marriage	0 to 4	1		1	
	5 to 9	**1.22**	1.04–1.43	1.23	0.99–1.52
	10 to 19	**1.26**	1.09–1.46	**1.35**	1.02–1.78
	20+	1.11	0.95–1.30	1.25	0.91–1.70
No. of living children	0	1		1	
	1 to 2	**0.84**	0.71–1.00	**0.80**	0.65–0.98
	3 to 4	**0.80**	0.67–0.95	**0.78**	0.63–0.97
	5+	**0.66**	0.54–0.80	**0.66**	0.51–0.84
Exo-system					
Place of residence	Urban	1		1	
	Rural	0.92	0.82–1.03	1.00	0.85–1.17
Caste or tribe	SC	1		1	
	ST	**0.82**	0.71–0.96	0.95	0.78–1.14
	OBC	0.99	0.87–1.11	0.93	0.81–1.07
	Other	**0.71**	0.62–0.80	**0.80**	0.69–0.94
Wealth Index	Poorest	1		1	
	Poorer	1.12	0.99–1.26	1.16	1.00–1.34
	Middle	1.11	0.98–1.26	1.11	0.95–1.30
	Richer	**1.15**	1.00–1.31	**1.28**	1.06–1.55
	Richest	1.00	0.85–1.18	1.21	0.94–1.56
Occupation	Not working	1		1	
	Agricultural	**1.21**	1.09–1.34	**1.14**	1.00–1.29
	Other	**1.32**	1.18–1.48	1.08	0.94–1.23
Macro-system					
Region	North	1		1	
	East	0.85	0.71–1.02	0.86	0.69–1.07
	Northeast	**0.71**	0.60–0.85	**0.67**	0.55–0.83
	West	0.89	0.72–1.09	0.93	0.73–1.17
	Central	1.16	0.99–1.36	1.09	0.90–1.32
	South	**1.71**	1.46–2.00	**1.37**	1.12–1.67
Religion	Hindu	1		1	
	Muslim	0.87	0.75–1.01	1.06	0.88–1.27
	Christian	1.11	0.91–1.35	**1.31**	1.00–1.71
	Other	1.21	0.99–1.48	**1.50**	1.20–1.87
Trans-system					
Thinks that violence can be justified	No	1		1	
	Yes	0.99	0.90–1.10	**0.89**	0.79–0.99

^a^Adjusted for all variables

Bold fonts denote significant (*p* < 0.05) crude OR and adjusted OR

Having been married for 5–9 years or 10–19 years, being in employment and being in the ‘richer’ wealth category were all associated with seeking help for spousal violence. Having children and belonging to a ST or other caste than SC and OBC were negatively associated with seeking help. There were also regional variations in help-seeking behaviour; women in the South were 1.71 times (95% CI 1.46–2.00) more likely to seek help than women in the North and women in the Northeast were even less likely to seek help. The variables place of residence, religion and attitude towards wife-beating did not predict help-seeking behaviour in the bivariate model.

### Predictors of help-seeking: Multivariate model

After controlling for all other variables, experience of severe violence and having been injured at least once were still the strongest predictors of seeking help although the odds ratios had declined to 2.80 (95% CI 2.50–3.15) and 2.79 (95% CI 2.49–3.14) respectively (Table 3). Women who had been at least 21 years old when they got married, women who had been married for 10–19 years and women who had completed primary education were more likely to seek help. Having children was a risk factor for not seeking help. Women with five or more children were 34% less likely to seek help for violence than those with no children.

Belonging to another caste than SC, ST and OBC, belonging to the second highest wealth quintile (‘richer’) and working in the agricultural sector were still predictors of help-seeking in the multivariate model. Living in the South region was a positive predictor of seeking help whereas living in the Northeast was a risk factor for not seeking help. In contrast with the results of the bivariate regression religion and attitude to wife-beating were predictors of help-seeking behaviour after controlling for other variables. Christian women and women who were adherents of other minority religions (usually Buddhism or Sikhism) were more likely to have sought help than Hindu women. A belief that wife-beating is justified in at least some circumstances was a risk factor for not seeking help.

Multivariate regressions showing factors associated with help-seeking for specific types of violence and combinations of violence are presented in Additional file 1: Table S1. Severity of violence, having been injured and region were predictors of help-seeking behaviour for all types of violence. The most noticeable result from these regressions was that women in the South were almost four and a half times more likely to seek help for a combination of physical and sexual violence than women in the North. Other examples of strong predictors of help-seeking were religion and education. In the case of women who had experienced physical, sexual and emotional abuse, women who had completed secondary education were twice as likely to seek help as women who had not completed primary education. Furthermore, adherents of minority religions were twice as likely as Hindus to seek help if they had experienced both physical and sexual violence or all three types of violence. When exposed to physical violence only, women in the middle three wealth quintiles (poorer, middle and richer) had higher odds of seeking help compared to women in the lowest wealth quintile. Having a father who had beaten one’s mother was a negative predictor of help-seeking in the multivariate model (OR: 0.76; 95% CI 0.62–0.93), but only for women who had experienced both physical violence and emotional abuse.

## Discussion

The results of this study indicate that the vast majority of women in India who are exposed to spousal violence do not seek help to stop it. Only 23.7% of the women who had experienced some form of spousal violence had sought help from someone. This figure is lower than that reported by Palermo et al. [35], who found that 32% of women exposed to gender-based violence had sought help from anyone. Gender-based violence is not limited to violence perpetrated by a husband or intimate partner. A comparison of the two different results therefore demonstrates that it is less common to seek help for violence when the perpetrator is a husband. The prevalence of reporting spousal violence to formal institutions in India was 1%, consistent with findings from other studies on help-seeking for spousal violence in India [14, 15]. The low proportion of women who seek help indicates that Indian society views violence within marriage as a family matter. It is expected that Indian women will be good and dutiful wives and mothers and will sacrifice themselves for their family; they are also expected not to disclose family matters to others [18]. This places them in a difficult position should their husband be violent. Some may choose to keep silent about their experience in order to maintain the façade of a good family. Andersson et al. suggested that Pakistani women’s economic dependence on their husband may contribute to their decision to remain silent [11]; similar factors are likely to be at work in India.

Our study also revealed that the prevalence of help-seeking in India varies considerably with the form of violence. Women who had experienced all three types of spousal violence (physical and sexual violence and emotional abuse) were most likely to seek help (48.8%). Women who were exposed to sexual violence only, or a combination of sexual violence and emotional abuse were least likely to seek help (1.5% and 6.2% respectively). The very low frequency at which help is sought to stop sexual violence reflects a culture and legal code in which a husband is considered entitled to his wife’s body. Marital rape is not a crime in India; this aspect of the legal code may influence attitudes to sexual violence within marriage, but equally the law may be considered to reflect societal attitudes to sexual violence within marriage.

### The ecological model

**Fig. 1:**
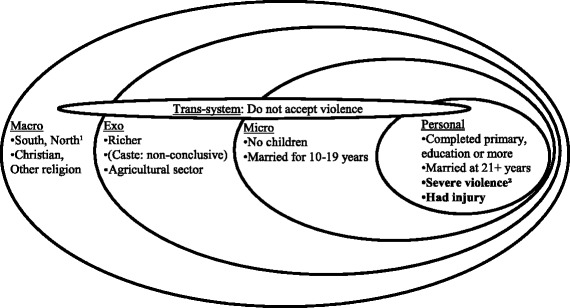
Factors that increased the odds of seeking help for spousal violence in terms of the ecological model for violence, redrawn from Heise (1998) [45]. An additional dimension, a transboundary system, is added for completeness

The findings from this study provide support for the core idea of the ecological model, that women’s help-seeking behaviour is influenced by factors at several levels (see Fig. 1). In this section we relate seeking help for spousal violence to the ecological model.

At the personal history-level the most important predictors of seeking help were experience of severe violence and having suffered physical injury as a consequence of spousal violence. Other studies have reported similar findings [12, 14, 17, 29, 31]. Also in line with other research, but contradictory to results from the study by Rowan et al. [14] we found that having more education increased the probability that a woman would seek help for spousal violence. Our results show that for women who had been exposed to both physical, sexual and emotional spousal violence, education was even a strong predictor of help-seeking. Having completed secondary or higher education doubled the odds of seeking help compared to not having education or not completed primary education. Education is generally believed to empower people and literacy enables women to access information, including information about the law, more easily.

Our study also found that age at first marriage is a predictor of help-seeking behaviour for spousal violence in India. In India the age gap between spouses is often larger in marriages where the girl was particularly young at the time of the marriage. Jensen and Thornton (2010) suggested that a large age gap between husband and wife has an impact on the woman’s power, position and autonomy within the family. Women who marry young also tend to receive less education [51]. It is reasonable to suggest that a woman who is older when she marries will be more mentally mature at the time of the marriage and will maintain greater autonomy and agency within her marriage as a result.

Few micro-system factors were examined in this study but the results indicate that in India having many children was a risk factor for not seeking help for spousal violence. In South Asia reporting IPV can have serious implications for a woman; she may lose her children or find herself unable to provide for them if she leaves her husband [11, 18]. These might be reasons for why women with children were less likely to seek help compared with women with no children.

Women’s socio-economic status, caste, and occupation were exo-system predictors of help-seeking behaviour, however the category ‘other caste’ was heterogeneous, so finding that women who belonged to ‘other castes’ were less likely to seek help than women who belong to a SC is not particularly informative. The fact that women in the middle three wealth quintiles (poorer, middle and richer) were more likely than the poorest women to seek help when exposed to physical violence only implies that having at least some material resources may make it easier for women to seek help. The poorest women may have no choice but to accept their situation and stay with their husband [24]. It is interesting to note that the richest women were not more likely to seek help than the poorest women. Tichy et al. (2009) suggested that in India higher status women are less likely to recognise IPV as a ‘societal problem’ and less likely to identify abuse than impoverished and working class women [52]. It is possible that wealth and prestige are associated with under-reporting of violence.

National and Indian regional differences in the probability of seeking help for spousal violence indicate that cultural factors and social norms influence women’s help-seeking behaviour. This indicates that the wider social and cultural context play an important role in help-seeking behaviour. It is likely that within India there are regional differences in societal acceptance of violence against wives, or the rigidity of gender roles. Jejeebhoy et al. (2013) discuss a North-South divide in India, where for example women in the South of India have more autonomy than their counterparts in the North and the East, and there is also tighter social control in the northern states [53]. There may be higher pressure to conform to the social norms in societies where tight social control prevails, causing more women to keep silent about spousal violence. There are also regional differences in the implementation of legal provisions regarding violence against wives [24]. The lack of legal sanctions against IPV and spousal violence in India may also contribute to reducing the proportion of women in India who seek help for spousal violence.

Religion was a predictor of help-seeking behaviour for almost all types of spousal violence, a result in line with findings from India and Nigeria [15, 33] but contradicts previous findings from Bangladesh [12]. In our study Christian women and women from other minority religious groups (except Muslim women) were more likely to seek help than Hindu women, perhaps because of the patriarchal and authoritarian structures of Hinduism.

As mentioned in the theoretical discussion of the ecological model, we found it difficult to place women’s personal attitude to violence in a single system. A woman’s attitude to violence might be considered an individual-level factor; however individuals’ attitudes are influenced by factors at various levels, for example by societal norms. To provide a more complete description of the context in which IPV occurs the ecological model should therefore be modified to incorporate trans-system factors relevant to all research on help-seeking for IPV (see Fig. 1). Contrary to Hayes and Franklin’s study on women’s help-seeking in India, we found that individual attitudes towards violence were associated with women’s decisions about seeking help. Thinking that spousal violence can be justified decreased the likelihood of seeking help. This result is consistent with data from Pakistan [11]. It can be argued that women who think that a husband is justified in hitting or beating his wife in at least some circumstances normalise violence against wives, and are less likely to seek help because they believe a husband has a right to be violent towards his wife.

### Recommendations for policy and practice

The findings from this study and other similar studies demonstrate that a very small proportion of women in India seek help from formal institutions. This calls for action to facilitate the possibility and decision to turn to formal sources for help. Family members, friends and neighbours can often provide temporary support in form of food and shelter, and sometimes also intervene [25, 54], but they are seldom able to help the woman to change her situation in a long term-perspective [54]. Although IPV occurs in a family setting it is important that violence is not viewed as a private matter. IPV should also be seen as a political issue; interventions are required at several levels. Ensuring that all children complete at least primary, preferably secondary, education could help to empower girls, which might increase future help-seeking rates. The long-term benefits of school enrolment for girls could be promoted through media campaigns targeting parents. The legal age for marriage for girls is 18 in India but child marriage is still prevalent. We found that early marriage is associated with less help-seeking for spousal violence compared to getting married at the age of 21 or older. This, along with a children’s rights perspective, advocates for efforts to prevent child marriages. India is already making progress. The proportion of Indian girls who get married before the age of 18 has declined from 47% in 2005–2006 [21] to 27% in 2015–2016 [22]. A further decline in the prevalence of child marriage would empower more girl. It would have a positive effect on many aspects of girl’s lives, including an increasing likelihood to seek help if exposed to spousal violence.

However, only focusing on interventions to improve women’s empowerment at the individual level may not alone result in a considerable increase in help-seeking as long as patriarchal norms accepting violence still prevail [14]. It is therefore crucial to also address norms and attitudes relating to violence, as well as to women who disclose violence. Awareness of the problem of IPV, as well as awareness of formal support services that victims of IPV can turn to, need to be raised among the public.

Apart from lacking knowledge of formal support services, women in India seem to lack trust in formal institutions such as the police. It is of great importance that women who seek help from these institutions are treated with respect, taken seriously and given appropriate help. Research witness of women being afraid to go to the police because they lack knowledge of the police and some even think that they will be arrested themselves. Perceptions that the police will tell the woman to modify her behaviour instead of trying to make the husband stop being violent seem to be common [26]. Attitudes to IPV among police officers need to be addressed and education in how to respond to IPV is necessary. The police also need to work on gaining trust from the general public.

Given the prevalence of IPV in India and the low help-seeking rates, it can be discussed whether women should be screened for violence during health care visits or not. As mentioned in the literature review, a study from Mumbai indicated acceptability of screening among the patients in the study since two thirds of them would be willing to disclose violence if asked about it in a health care setting [25]. There is no consensus on whether screening for IPV in health care settings is beneficial or not, but there seem to be a predominant view in the literature, including WHO guidelines, is that universal screening for IPV cannot be justified. Several studies have found that universal screening for IPV did not decrease the recurrence of violence or improve the health of IPV victims [55, 56]. In settings where prevalence of IPV is high and referral options limited, the capacity to respond to victims of IPV identified through screening is likely to be insufficient. Instead selective enquiry may be more beneficial to victims of IPV [55]. Health care providers should ask about IPV when assessing conditions that may be caused by IPV, in accordance with WHO recommendation. However, evidence from India suggests that health care providers were reluctant to asking patients about IPV, especially sexual IPV [57]. Barriers to asking patients about IPV perceived by obstetricians in Pakistan included not having a solution to the problem, feeling uncomfortable discussing the subject with patients and fear of police involvement for the obstetricians themselves [58]. Similar barriers are likely to be perceived by health care providers in India. This point to the importance of training of health care staff in order for them to overcome reluctance to discussing IPV with patients. We recommend that efforts within the health care system should focus on training health care providers in how to respond to women who disclose violence.

In the meantime it is also important to take measures to further reduce and prevent violence against women. Discussions about gender equality and gender roles should be part of the educational curriculum as early as in primary school. Norms and values should also be targeted through community-based interventions where local political and religious leaders should be involved to increase the acceptability and impact of such interventions. It is of great importance that boys and men are included and targeted in efforts for a more gender equal society.

By revealing that the proportion of women who seek help from someone when exposed to sexual spousal violence is almost non-existent in India, our study demonstrates that sexual spousal violence is, even more so than physical and emotional spousal violence, viewed as a private matter. The legal framework could strengthen women’s rights and also influence people’s attitudes towards rape within marriage by defining rape within marriage as a crime.

Further research is needed on the help-seeking behaviour of Indian women. Research into what could make women in India more likely to report violence to formal institutions would provide valuable information which could be used to inform policy and improve services for women who experience IPV. Furthermore, attitudes to IPV among the police force and judicial system in India should be studied.

### Methodological considerations

The cross-sectional nature of the data was a limitation of this study. Age, number of children and marital duration may have been different when the women experienced spousal violence. The operationalisation of help-seeking was another limitation. The question about help-seeking behaviour related only to help to stop the violence. Data on leaving a partner without seeking help was not captured so women who left their partner without seeking help were classified as non-help-seekers. Due to the sensitive nature of the questions asked to the respondents in the survey it is likely that not all women who had been exposed to spousal violence reported that they had – a problem that is always prevalent when conducting research on IPV and spousal violence.

Because the question about help-seeking behaviour related to all violence the woman had experienced over their lifetime we excluded women who had experienced violence from someone other than their husband from our analysis. This means that our findings relate only to women who have experienced spousal violence but no other form of violence; they may not generalise to women with wider experience of violence. Data on whether the respondent had experienced violence repeatedly or only on one occasion was available only for women who had experienced violence in the 12 months before the survey, so this variable was not included in the analysis although it is likely to be an important predictor of help-seeking behaviour. In addition, multilevel analysis would have been a better approach to investigating macro-system variables. Although variables were allocated to the different levels of Heise’s model following the literature when possible [18, 45, 46], we are aware that other conceptualizations are also possible. For instance, wealth was considered as an exo-system variable in our study but it could also have been included as a trans-system variable. While estimates in our model would not be modified, the interpretations of the results could have been different. The data were collected in 2005–2006 which means that our findings may not reflect the current situation.

Although the data were collected in 2005–2006 we believe this study provides an indication of how unlikely it still is that women in India who experience spousal violence will seek help, as well as information about factors associated with seeking help.

## Conclusions

In summary, this study suggests that the rate at which help is sought for spousal violence in India is low, and that the proportion of women seeking help from formal institutions is almost non-existent. This study also considered how help-seeking behaviour varies with the type of violence or abuse. Help-seeking was most common among women who experienced all forms of spousal violence and rarest among women who experienced sexual violence only.

The ecological model does not explain the process of help-seeking, but it describes the decision to seek help in terms of interactions among multiple factors at multiple levels. Variables at all levels of the ecological model were associated with seeking help for spousal violence. However, it seems that for women in India, factors at the personal history-level and macro-system level were the most important predictors of help-seeking behaviour. Experience of severe violence and having had an injury as a consequence of the violence were the two strongest correlates of seeking help. Implications of this study include the need for national and local efforts to increase gender equality and to change norms and attitudes towards IPV as well as to women who seek help for IPV.

## Additional file

Additional file 1:
**Table S1.** Table with results from multivariable logistic regression analyses for predictors of seeking help for different types of spousal violence (XLSX 18 kb)

## Abbreviations

CI: Confidence interval
IPV: Intimate partner violence
NFHS: National Family Health Survey
NGO: Non-Governmental Organization
OBC: Other backward class
OR: Odds ratio
PWDVA: Protection of Women Against Domestic Violence Act
SC: Scheduled caste
ST: Scheduled tribe
WHO: World Health Organization
